# Plasma and Urinary (Poly)phenolic Profiles after 4-Week Red Raspberry (*Rubus idaeus* L.) Intake with or without Fructo-Oligosaccharide Supplementation

**DOI:** 10.3390/molecules25204777

**Published:** 2020-10-17

**Authors:** Xuhuiqun Zhang, Amandeep Sandhu, Indika Edirisinghe, Britt M. Burton-Freeman

**Affiliations:** Department of Food Science and Nutrition and Center for Nutrition Research, Institute for Food Safety and Health, Illinois Institute of Technology, Chicago, IL 60616, USA; xzhan198@iit.edu (X.Z.); iedirisi@iit.edu (I.E.); bburton@iit.edu (B.M.B.-F.)

**Keywords:** red raspberry, fructo-oligosaccharide, prediabetes, urolithins, phenyl-γ-valerolactones, phenolic acids, UHPLC-QQQ

## Abstract

Red raspberries (RRB) are high in anthocyanin- and ellagitannin- type (poly)phenols. This study aimed to investigate the effect of 4-week RRB supplementation on (poly)phenolic metabolism in adults with prediabetes and insulin-resistance (PreDM-IR); and whether adding fructo-oligosaccharides (FOS), prebiotics, would augment the microbial metabolites of RRB (poly)phenols. In a randomized crossover clinical trial, subjects (n = 35: PreDM-IR, n = 25; healthy Reference group, n = 10) consumed 1 cup RRB (fresh weight equivalence) per day and RRB with 8 g FOS per day each for 4 weeks in random order separated by 4-week washout. Plasma and urinary (poly)phenolic metabolites were characterized after (0–24 h) consuming a RRB-based test drink (2 cups RRB) at baseline/week 0 and again after 4-week supplementations. A total of 123 (poly)phenolic metabolites were quantified. After 4-week RRB supplementation, several metabolite groups were significantly increased (*p* < 0.05), including urolithins, phenyl-γ-valerolactones, and phenolic acids. Supplementing FOS with RRB for 4 weeks enhanced benzoic acid derivatives compared to the baseline (*p* < 0.05). Specific effects of supplementation by metabolic status indicated 4-week RRB supplementation significantly increased microbial metabolites that were lower in PreDM-IR group. Our results suggest alterations in the capacity of PreDM-IR group to metabolize and render bioavailable raspberry-derived (poly)phenols when consumed regularly.

## 1. Introduction

Red raspberries (*Rubus idaeus* L.) contain appreciable amounts of bioactive (poly)phenol compounds, particularly anthocyanins, ellagitannins and flavan-3-ols [1,2]. Whole red raspberries (RRB) and their (poly)phenol components have been associated with metabolic health, including glucose control, increasing insulin sensitivity, and improving lipid profiles in humans, and reducing fat mass in animal studies [3,4]. Some of these (poly)phenols could be absorbed intact, while majority of them are degraded to products of gut microbial metabolism [5,6,7]. For example, the majority of ellagitannins and oligo- and poly-meric flavan-3-ols escape proximal gastrointestinal (GI) tract absorption and are catabolized in the colon to urolithins and phenyl-γ-valerolactones, respectively, by gut microbiota [7,8]. Inter-individual differences and putative metabotypes of these microbial metabolites have been observed, but their association with metabolic health is not fully understood [8,9].

Fructo-oligosaccharides (FOS) are soluble dietary fibers, and as prebiotics, they are selectively fermented by specific gut microbiota [10]. FOS are naturally present in select fruits and vegetables or produced from beet sugar, frequently used to replace sugars in the formulation of low-sugar food [11]. Animal studies indicate FOS augments (poly)phenolic metabolites generation: adding FOS to a 4-week intake of (poly)phenol concentrate from apple pomace enhanced the hydrolysis of quercetin 3-*O*-glucoside in rats [12]; and adding FOS to a 4-week strawberry feeding study enhanced urolithins production in rats [13].

Prediabetes (PreDM) is an intermediate stage in the development of type 2 diabetes mellitus (T2DM) that usually co-occurs with a cluster of metabolic risk factors, e.g., insulin resistance, dyslipidemia, hypertension, visceral obesity, and elevated inflammatory markers, leading to substantially increased risk of T2DM and cardiovascular diseases [14]. The Global Burden of Disease study identified low intake of fruit among the top three dietary risks for cardiovascular disease and T2DM [15]. The mechanisms underlying this finding are unknown. Recent studies have observed aberrant gut microbiome structure in PreDM individuals [16,17]. One possible explanation is individuals with PreDM have diminished microbial capacity to metabolize fruit bioactive compounds, such as (poly)phenols, to their respective bioavailable metabolites. It is unclear if regular exposure to fruit (poly)phenols or a combinatorial regimen of fermentable fibers with a (poly)phenol rich fruit would enhance or alter the metabolite milieu.

**Figure 1:**
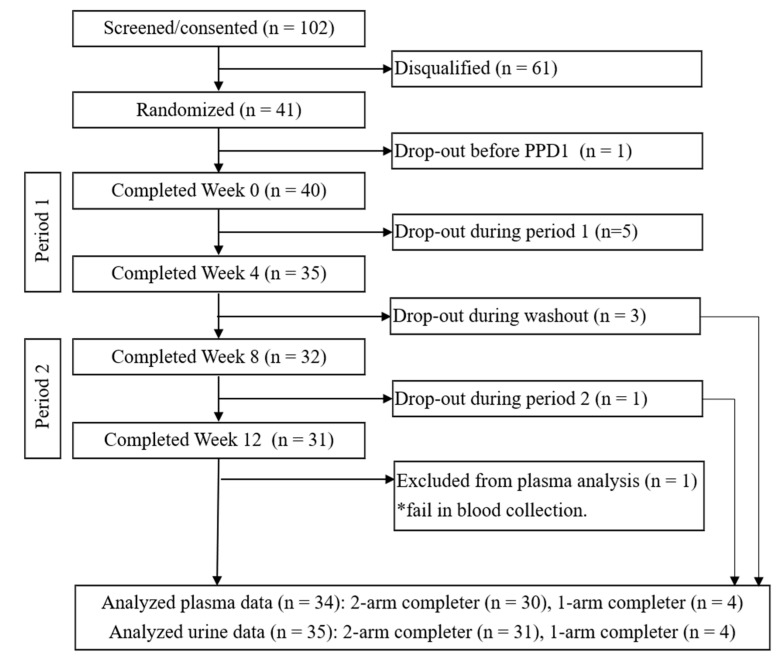
Consolidated Standards of Reporting Trials (CONSORT) flow diagram of the study.

**Figure 2:**
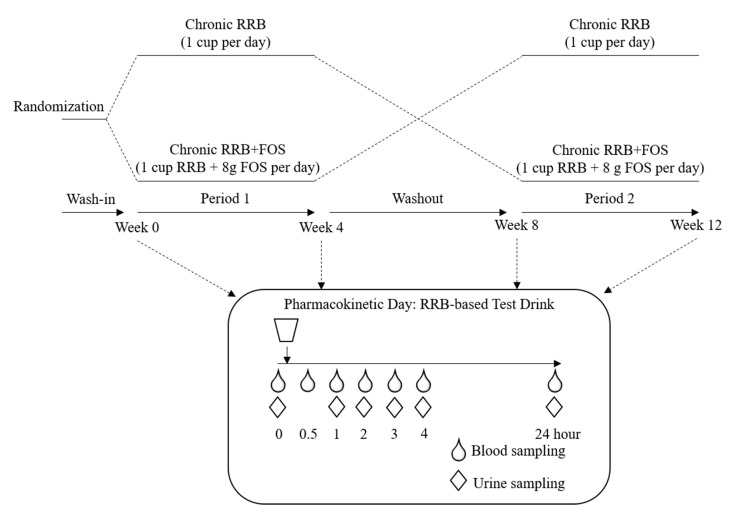
Study design and pharmacokinetic day schema.

Therefore, the present study aimed to investigate the effect of supplementing RRB with or without FOS on (poly)phenol metabolism for 4 weeks in at risk group of individuals with prediabetes and insulin resistance (PreDM-IR). Metabolically healthy individuals served as a Reference control group. Our working hypothesis is that the PreDM-IR group will have a distinct (poly)phenolic metabolites profile and supplementing their diets with RRB for 4 weeks will increase microbial-derived (poly)phenolic metabolites and adding FOS will augment the effect. This study was a randomized, single-blinded, two-arm, 4-week, within subject crossover design incorporating a modified pharmacokinetic (PK) multi-sampling protocol with a RRB-based test drink (RRBtest) to assess RRB (poly)phenolic metabolism before and after supplementing the diet for 4 weeks with RRB and RRB+FOS (Figures 1 and 2).

## 2. Results

### 2.1. Subject Demographics and Characteristics

A total of 102 subjects were screened, out of which 41 passed the initial screening and returned for randomization (Figure 1). Ten participants withdrew from the study, six during period 1 and four during period 2, because of inability to comply with study procedures, such as schedule conflict and lack of follow-up. One subject was excluded from the plasma analysis due to failure in blood collection. The analyzed plasma data set included 34 men and women, PreDM-IR (n = 24) and Reference (n = 10). The evaluable urine data set included 35 men and women, PreDM-IR (n = 25) and Reference (n = 10). The PreDM-IR and Reference groups underwent the same study procedures (Figure 2). No adverse events related to supplementations were reported. Demographic characteristics are shown in Table 1.

### 2.2. (Poly)phenol Content of RRB Interventions

A total of 44 (poly)phenols were quantified in the different RRB drinks, including 6 anthocyanins, 17 ellagitannins, 10 flavan-3-ols, 6 flavonols and 5 phenolic acids (Table 2). The RRBtest drink contained 388.4 ± 3.3 mg (poly)phenols, including 61% anthocyanins, 32% ellagitannins, 5% flavan-3-ols, 1% flavonols and 1% phenolic acids. The daily RRB drink (1 cup fresh equivalence) contained 129.7 ± 1.9 mg (poly)phenols, including 60% anthocyanins, 33% ellagitannins, 4% flavan-3-ols, 1% flavonols and 2% phenolic acids. The daily RRB+FOS drink contained 129.8 ± 0.5 mg (poly)phenols, including 60% anthocyanins, 33% ellagitannins, 5 % flavan-3-ols, 1% flavonols and 2% phenolic acids.

### 2.3. Plasma and Urine (poly)phenolic Metabolites: Effect of Chronic Exposure to RRB or RRB+FOS

A total of 123 (poly)phenolic metabolites were quantified in plasma and urine samples, classified as anthocyanin, urolithin, phenyl-γ-valerolactone and phenolic acid derivatives. Phenolic acid derivatives were further classified into benzaldehyde, cinnamic acid, phenylpropionic acid, phenylacetic acid, benzoic acid and hippuric acid derivatives (Table S1).

**Figure 3:**
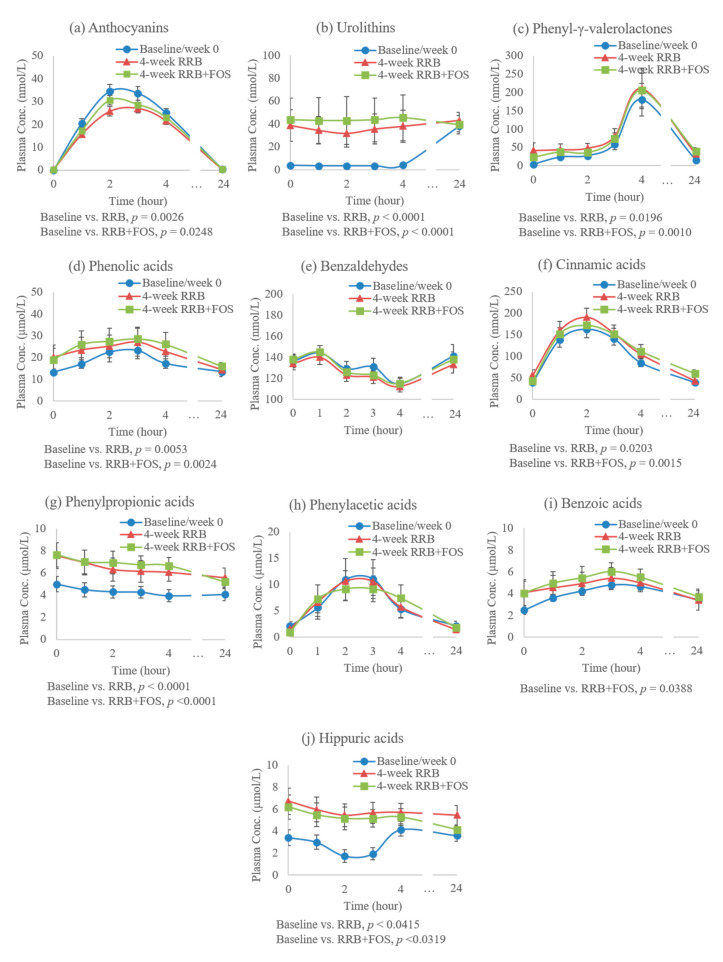
(Poly)phenolic metabolites concentrations in human plasma (0–24 h) post-consumption of RRBtest drink at the baseline/week 0, after 4-week RRB and 4-week RRB+FOS supplementations: (**a**) total anthocyanin derivatives; (**b**) total urolithin derivatives; (**c**) total phenyl-γ-valerolactone derivatives; (**d**) total phenolic acid derivatives; (**e**) total benzaldehyde derivatives; (**f**) total cinnamic acid derivatives; (**g**) total phenylpropionic acid derivatives; (**h**) total phenylacetic acid derivatives; (**i**) total benzoic acid derivatives; and (**j**) total hippuric acid derivatives.

Figure 3 illustrates the pharmacokinetic (PK) profile of metabolites in response to RRB test drink at the baseline (week 0) when subjects were following their usual diets (low in fruit and vegetables and devoid of berries) as well as after 4-week RRB and RRB+FOS supplementations in diets.

Compared to baseline, consuming RRB daily resulted in significantly reduced concentrations of total anthocyanin derivatives, and increased total urolithins, phenyl-γ-valerolactones and select phenolic acid derivatives, i.e., cinnamic acid, phenylpropionic acid and hippuric acid derivatives, in plasma compared to baseline/week 0 (n = 34, *p* < 0.05, Figure 3). Adding FOS to 4-week RRB regimen had limited influence on metabolite concentrations. Only the benzoic acid derivatives were significantly increased from baseline when FOS was added to RRB (*p* = 0.0388, Figure 3).

**Figure 4:**
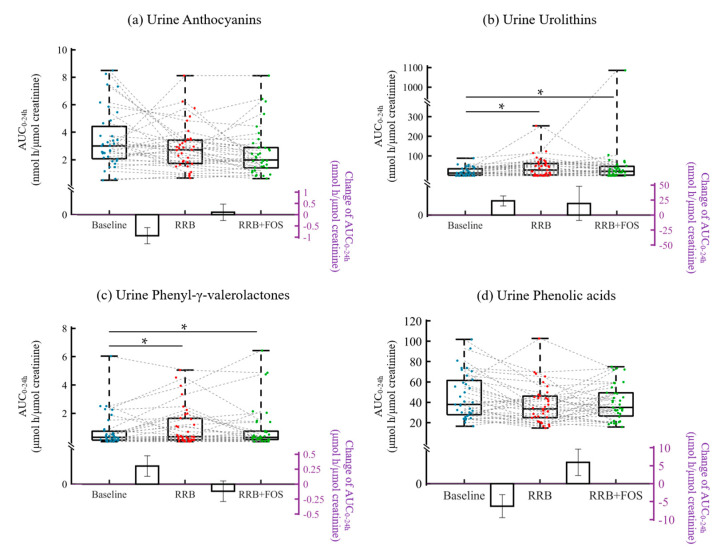
Urinary (poly)phenolic metabolites AUC_0–24h_ (areas under the 24-h curve) post-consumption of RRBtest drink at the baseline/week 0, after 4-week RRB and 4-week RRB+FOS supplementations: (**a**) total anthocyanin derivatives; (**b**) total urolithin derivatives; (**c**) total phenyl-γ-valerolactone derivatives; (**d**) total phenolic acid derivatives. * Significant supplementation effect, *p* < 0.05. The box and whisker plots illustrate the distribution of values for each study visit. The line in the middle of the box is plotted at the median, the inferior and superior limit of the box correspond to the 25th and the 75th percentiles, respectively. Bars represent mean change of AUC_0–24h_ at baseline vs. 4-week RRB and 4-week RRB vs. 4-week RRB+FOS, with their standard errors.

In urine, 4-week RRB supplementation resulted in significantly increased total urolithin and phenyl-γ-valerolactone derivatives (*p* < 0.05), while adding FOS to 4-week RRB did not affect the metabolite excretion concentrations (Figure 4). Supplementations did not affect body composition or vital signs (data not shown).

### 2.4. Metabolic Status and Plasma and Urine (Poly)phenolic Metabolites

**Figure 5:**
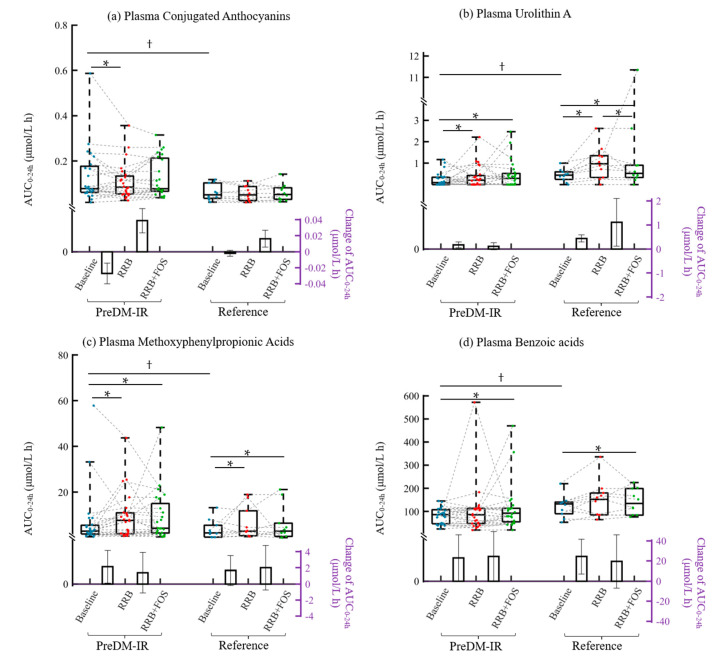
Effect of 4-week RRB and RRB+FOS supplementations on the (poly)phenolic metabolites between PreDM-IR and Reference groups: (**a**) plasma conjugated anthocyanins; (**b**) plasma urolithin A derivatives; (**c**) plasma methoxyphenylpropionic acid derivatives; and (**d**) plasma benzoic acid derivatives. ^†^ Significant Group difference at the baseline, *p* < 0.05; * Significant supplementation effect within a group, *p* <0.05. The box and whisker plots illustrate the distribution of values within each group for each study visit. The line in the middle of the box is plotted at the median, the inferior and superior limit of the box correspond to the 25th and the 75th percentiles, respectively. Bars represent mean change of AUC_0–24h_ at baseline vs. 4-week RRB and 4-week RRB vs. 4-week RRB+FOS per group, with their standard errors.

At the baseline, PreDM-IR group had significantly higher plasma concentrations of conjugated anthocyanin derivatives and methoxyphenylpropionic acid derivatives and lower concentration of urolithin A derivatives and benzoic acid derivatives compared to the Reference group (*p* < 0.05) (Figure 5). Metabolic status was a factor in metabolite responses to the 4-week supplementations. Compared to the baseline, 4-week RRB supplementation resulted in significantly decreased conjugated anthocyanin concentrations and increased urolithin A and methoxyphenylpropionic acid derivatives in the PreDM-IR group (*p* < 0.05) (Figure 5). Adding FOS to the RRB attenuated the effect of RRB in reducing conjugated anthocyanin concentrations, and significantly increased benzoic acid derivatives in the PreDM-IR group. In the Reference group, adding FOS to RRB further increased urolithin A production compared to RRB supplementation alone (*p* = 0.0074) (Figure 5b).

## 3. Discussion

This is the first study to report the profile of (poly)phenolic metabolites, i.e., parent, phase II, and gut microbial metabolites of RRB (poly)phenols, on a berry/red raspberry devoid background diet and compared to a diet supplemented with RRB or RRB+FOS for 4-weeks in at risk group of individuals with PreDM-IR. The main findings of the research are summarized: (1) 4-week RRB supplementation increased microbial-derived metabolites of RRB (poly)phenols, e.g., urolithins, phenyl-γ-valerolactones and select phenolic acids, and addition of FOS to the RRB regimen had limited influence except for enhancing benzoic acids derivatives compared to the baseline/week 0 (*p* < 0.05); (2) 4-week RRB supplementation significantly reduced conjugated anthocyanin derivatives, which were significantly higher in the PreDM-IR group, and increased urolithin A and benzoic acid derivatives, which were significantly lower in the PreDM-IR group compared to the Reference group (*p* < 0.05).

Anthocyanins accounted for approximately 61% of total RRB (poly)phenols (Table 2). After RRB intake, some parent anthocyanins can be absorbed intact in their glycosylated form, while others may be hydrolyzed to their aglycones, and both forms could be subjected to phase I and II metabolism in the small intestine, liver, and/or kidney, forming methyl, glucuronide, or sulfate conjugated metabolites [5,18]. In this study, all five parent RRB anthocyanins were detected in plasma and urine samples post-consumption of RRBtest drink. Methyl and glucuronide conjugated parent and aglycone anthocyanins were also found in plasma and urine samples. The concentration of anthocyanin derivatives in plasma were comparable with those observed by de Ferrars et al. after ingestion of 500 mg [^13^C_5_]cyanidin-3-*O*-glucoside, serum AUC_0–24h_ of total anthocyanin around 279 ± 170 nmol/L h [19]. However, another RRB clinical trial (300 g frozen RRB) reported only 0.007% anthocyanin in plasma (much lower than the present study), which may be caused by the exclusion of cyanidin 3-*O*-sophoroside in quantification, the most abundant anthocyanin in both RRB and biological samples after RRB intake [1]. It was noticeable that the PreDM-IR group had significantly high concentrations of total conjugated anthocyanins (~45%) compared to the Reference group at the baseline, and the 4-week RRB supplementation substantially decreased the concentration of conjugated anthocyanins (~27%) (Figure 5a). The mechanism underlying the variations observed in metabolite concentrations in PreDM-IR and Reference groups is not clear. However, the reduction of conjugated anthocyanins could be associated with the metabolic health status of individuals that needs further research.

Ellagic acid and ellagitannins constituted around 32% of total RRB (poly)phenols (Table 2). After RRB intake, some ellagitannins are hydrolyzed to ellagic acid during gastric transit, but their parent forms are poorly absorbed by the proximal GI tract. In the colon, gut microbiota, e.g., *Gordonibacter urolithinfaciens* and *Ellagibacter isourolithinifaciens*, participate in the transformation from unabsorbed ellagitannins to various urolithins, including three final forms, i.e., urolithin A, urolithin B and isourolithin A, which are further subjected to phase II metabolism and suggested to exert biologically active effects [9,20,21]. In this study, urolithin A, urolithin B, isourolithin A and their conjugated metabolites were identified in plasma and urine, however, not all the subjects were able to produce urolithins and inter-individual variations were observed (Figure 4 and Figure 5). Human intervention studies with ellagitannin-rich foods, e.g., pomegranate, strawberries and walnuts, have illustrated the inter-individual variability in ellagitannin metabolism. Based on the data, individuals were classified as three different urolithin metabotypes (UMs): urolithin metabotype A (UM-A) is distinguished by the production of urolithin A; in urolithin metabotype B (UM-B) individuals produce isourolithin A and urolithin B besides urolithin A, and those with metabotype 0 (UM-0) do not produce these final urolithins [9,22]. At the baseline, 10 individuals of the PreDM-IR group (n = 25) qualified as UM-A, 10 qualified as UM-B and 5 as UM-0. In the Reference group (n = 10), 5 qualified as UM-A, 4 as UM-B and 1 as UM-0 subjects. After the 4-week RRB supplementation, one UM-0 in PreDM-IR group became UM-A. As is shown in the current study (Figure 3b), the 4-week RRB or RRB+FOS supplementation resulted in increased fasting (0 h) urolithin concentrations (~8 fold) compared to the fasting concentrations at baseline week 0, but no further increase was observed 24 h after the RRBtest drink as was observed at baseline 24 h. The increased excretion of urolithins was observed in 0-4 h urine samples while their excretion remained unchanged at 24 h. These data suggest that supplementation with RRB with or without FOS may contribute to the accumulation of urolithins but may not change the maximum concentration of urolithins.

Flavan-3-ols accounted for 5% of RRB (poly)phenols, mainly proanthocyanidin B dimers (Table 2). A majority of the ingested polymeric favan-3-ols are not absorbed in the upper part of the GI tract, but reach the colon, where the gut microbiota catabolize proanthocyanidins to phenyl-γ-valerolactones by A-ring fission; and subsequently, to phenylpropionic acids, phenylacetic acids and benzoic acids as the final microbial metabolites [7,23]. At the baseline, post consumption of the RRBtest drink, conjugated mono-, di- and tri- hydroxyphenyl-γ-valerolactones were quantified in plasma and urine, with AUC_0–24h_ 2.2 ± 0.5 μmol/L h and 0.8 ± 0.2 μmol h/μmol creatinine, respectively. Plasma C_max_ of total phenyl-γ-valerolactone derivatives (180 ± 44 nmol/L) was reached at 4 h after RRBtest intake. These findings are in accordance with a previous study on cranberries that reported plasma phenyl-γ-valerolactone derivatives C_max_ around 300 nmol/L at 4 h post-consumption of cranberry juice containing 787 mg (poly)phenols [24]. The plasma C_max_ of phenyl-γ-valerolactone derivatives are usually achieved at 4 to 6 h after consuming proanthocyanidin-rich foods, such as cranberry, tea and cocoa, and remain constant for several hours [8,25,26]. Bioactivity of phenyl-γ-valerolactones have been investigated both in vitro and in vivo, suggesting their role in regulation of inflammation, inhibition of cancer cell proliferation, reduction of systolic blood pressures, reduction of fat accumulation and prevention of urinary tract infections [7,27,28,29,30]. Thus, the significantly increased plasma and urinary concentration of phenyl-γ-valerolactones after a 4-week RRB and RRB+FOS intervention may result in associated health benefits.

Unabsorbed anthocyanins and phenyl-γ-valerolactones may be metabolized to various phenolic acids [5,6,7]. The microbial catabolism of anthocyanin is performed by cleavage of the heterocyclic flavylium ring (C ring fission), followed by microbial dehydroxylation or decarboxylation [5,6]. The different T_max_ of various subgroups of phenolic acids indicated a potential pathway for the catabolism of anthocyanins (Figure 3), such as: anthocyanins (2 h) → benzaldehydes (1 h) → cinnamic acids (2 h) → phenylpropionic acids (no peak time) → phenylacetic acids (2–3 h) → benzoic acids (3–4 h) → hippuric acids (4–24 h). Since the peak time of phenylpropionic acids wasn’t observed, their role in anthocyanin catabolism could be due to hydrogenation of cinnamic acids [1]. After the 4-week RRB or RRB+FOS supplementation, the plasma concentration of total cinnamic, phenylpropionic, and hippuric acids derivatives were enhanced (Figure 3), indicating the beneficial effect of RRB on the activity and/or abundance of gut microbiota responsible for (poly)phenol catabolism. Similarly, regular blueberry intake for 30 days increased benzoic and hippuric acid derivatives in human subjects [24]. Adding FOS to RRB alone augmented the effect on benzoic acids derivatives (Figure 3), possibly due to prebiotic fermentation by gut microbiota [10]. Besides conjugated anthocyanins and urolithins, metabolic health status of study participants also affected the phenolic acid profile. The PreDM-IR group had significantly lower benzoic acids derivatives (~21%) in plasma compared to the Reference group (Figure 5d), which was increased after the 4-week RRB+FOS supplementation (~11%). This may be attributed to the feeding effect of RRB (poly)phenols and/or prebiotics to gut microbiota that improved the capacity of gut microbiota in producing more phenolic acids.

This study is innovative in its focus on a group of people at risk for T2DM. The study design included a metabolically healthy Reference group to assess relevance of changes in the PreDM-IR group, which few other human studies include, however sample size of the Reference group was a limitation. It is noticeable that subjects within each group had different capacity to catabolize (poly)phenols and render metabolites, especially urolithins and phenyl-γ-valerolactones. Considering the sample size, inter-individual variations may limit the generalizability of conclusions. The RRBtest drink contained a bolus amount of RRB (2 cups of single-cultivar of RRB), while the 4-week supplementations contained 1 cup of multi-cultivar RRB. A limitation was the variance in RRB used in the test drink and those used for the 4-week supplementation. However, this is also valuable because the bolus amount of RRB (poly)phenols and single-cultivar RRB in the RRBtest drink enables the comparison with our previous acute study [31], and the RRB in the 4-week supplementation is consistent with typical intake of multiple RRB varieties in marketplace. Additionally, the amount (1 cup/d) was practical for participants to consume daily. Negative and positive treatment controls were not included in the study design due to subject burden limitations of a crossover clinical trial design. A crossover design was chosen for this discovery research to enhance power with a smaller number of subjects, as each serves as their own control. Plasma and urine samples were collected at 0–4 h and 24 h after the RRBtest drink to focus on anthocyanins and urolithins. However, collection of plasma and urine samples between 4–10 h may provide more PK information on other metabolites, which will be considered in future studies. Most phase II metabolites were quantified using their available parental compounds due to limited availability of metabolite standards (Table S1).

## 4. Materials and Methods

### 4.1. Study Design

This study was approved by the Institutional Review Board of Illinois Institute of Technology (IIT), Chicago, Illinois and registered with ClinicalTrials.gov (NCT03049631). All subjects provided written informed consent before initiation of any study procedures. The clinical part of study was conducted from May 2017 to February 2018 in the clinical unit of the Center for Nutrition Research (CNRC) at the IIT, Chicago, Illinois.

This study was a randomized, single-blinded, two-arm, 4-week, within subject crossover design incorporating the multi-sampling RRB-based test drink (RRBtest) protocol to assess RRB (poly)phenolic metabolism before and after supplementing the diet for 4 weeks with RRB and RRB+FOS (Figure 1 and Figure 2). Subjects were randomly assigned a supplementation order (RRB → RRB+FOS or RRB+FOS → RRB) separated by a 4-week washout period. Subjects were involved actively in the study for ~14 weeks from screening to completion. During each supplementation period, subjects came to the CNRC weekly to pick up standardized pre-made frozen drinks for daily consumption. At the end of each 4-week period, subjects participated in a “Pharmacokinetic Day (PKD)” visit following an identical protocol as baseline (week 0). Briefly, at each PKD, subjects consumed a RRBtest drink to access the PK profile of RRB (poly)phenols and their metabolites over 24 h. PK profiles after 4-week RRB or RRB+FOS supplementation were compared to baseline data. Each PKD lasted approximately 4.5 h with blood samples collected at 0, 1, 2, 3 and 4 h, urine sample collected at 0, 1, 2, 3 and 4 h, and then a final blood and urine sample the next morning (24 ± 1 h) calculated from the time of the RRBtest drink the day prior.

### 4.2. Study Participants

Men and women 20 to 60 years old were recruited from the greater Chicago land area and screened for participation at the CNRC/IIT. Subjects were required to meet general eligibility criteria and specific criteria for PreDM-IR. Specific PreDM-IR criteria included impaired fasting glucose (≥5.6 mmol/L and <7.0 mmol/L), elevated fasting insulin (>50th percentile cutoff) [32], and insulin resistance measured by homeostatic model assessment of insulin resistance, HOMA-IR ≥ 2 [31]. The Reference/Control group had fasting glucose values ≤ 5.6 mmol/L and a HOMA-IR value ≤ 1 [31]. General eligibility criteria for individuals to participate in the study were: non-smoker and not taking any medications that would interfere with outcomes of the study (i.e., lipid-lowering, anti-inflammatory, or glucose-interfering medications or dietary supplements), no known allergy or intolerance to berries, did not consume ≥3 servings of berries per week, and had no documented atherosclerotic disease, inflammatory disease, –GI or kidney disease, diabetes mellitus, or other systemic diseases. Women who were pregnant or lactating were not eligible to participate.

### 4.3. Study Foods and RRB Supplements

PKD visit: Foods consumed during a PKD visit included the RRBtest drink and fixed lunch and dinner meals. The RRBtest drink contained 250 g (~2 cups fresh weight equivalence) Individually Quick Frozen (IQF) RRB (*Rubus idaeus* L. var. Wakefield, Enfield Farms, Lynden, WA, USA) and 65 g dextrose (Table 1 and Supplementary Table S1). The 250 g RRBtest drink was chosen based on our prior research [31] and aims to deliver a bolus amount of red raspberry (poly)phenols to characterize (poly)phenolic metabolites over a 24 h period in plasma and urine. Subjects were asked to consume fixed meals provided by the CNRC the night before and on each PKD. One of the following three meals were offered, recorded, and maintained the same throughout the study: homemade meal, Jimmy John’s or Subway, or a meal prepared at CNRC (based on subject’s energy requirements).

4-week supplementation periods: The daily RRB drink for 4-week supplementation contained 50 g IQF RRB and 8 g freeze-dried RRB powder (multiple varieties, Van Drunen Farms, Momence, IL, USA). The daily RRB+FOS supplementary drink contained 50 g IQF RRB, 8 g freeze-dried RRB powder and 8 g short-chain FOS (Ingredion, Westchester, IL, USA). The 4-week RRB and RRB+FOS drink were designed based on practical dietary recommendation, incorporating 1 cup of berries per day, to understand the effect of regular fruit intake. The nutrient compositions of daily RRB and RRB+FOS supplementary drinks are listed in Table S2.

### 4.4. Study Procedures

Upon enrollment into the study, subjects had a diet stabilization period before starting the research protocol, which started with a low-(poly)phenol diet 3 days before the each PKD visit. In between PKD visits, subjects were counseled to keep low-berry diet (<3 servings of berries per week, except the experimental drinks), and maintained their usual dietary and physical activity patterns. Dietary intake data (24 h recalls) were collected and analyzed using the Automated Self-Administered 24 h (ASA24) Dietary Assessment Tool, version (2017), developed by the National Cancer Institute (Bethesda, MD, USA) [33]. Compliance to the diet and lifestyle restrictions was determined via ASA24 and via interview once per week. A fixed dinner meal was provided on the day before each PKD visit and the night of PKD visits to maintain consistency for the 24 h follow-up visit.

Subjects arrived at the CNRC in a fasting state (10–12 h, confirmed by finger stick) and well hydrated on the morning of each scheduled PKD visit. After assessing PKD readiness based on protocol compliance (i.e., dietary restrictions and fasting), anthropometrics, body composition and vital signs were measured. An intravenous catheter was placed in subjects’ non-dominant arm by a licensed health care professional. Fasting blood and urine samples were collected (0 h), and subjects were provided with the RRBtest drink. Subsequently blood samples were collected at 0.5, 1, 2, 3, 4, and 24 h and urine samples were collected at 1, 2, 3, 4, and 24 h. After the 4-h blood collection, the catheter was removed and subjects were evaluated for safety before leaving the CNRC. Subjects consumed the fixed lunch and dinner meals at home and returned the next morning (fasted, well hydrated) for the 24 h blood and urine collection to complete the PKD visit. Females of reproductive age were studied avoiding menstruation phase of their menstrual cycle.

### 4.5. Dietary Assessment

During the supplementation periods, ASA24 was completed by subjects once per week to record all food and drink consumed in the past 24 h. The total calorie, fat, protein, carbohydrate, sugar, fiber, vegetable amount, and fruit amount were calculated based on the ASA24 reports and analyzed with SAS 9.4 (SAS Institute, Inc., Cary, NC, USA). Background diets did not change significantly during the study periods (Table S3).

### 4.6. Chemical and Reagents

Standards of cyanidin 3-*O*-glucoside, cyanidin 3-*O*-rutinoside, pelargonidin 3-*O*-glucoside, pelargonidin 3-*O*-rutinoside, peonidin 3-*O*-glucoside, malvidin 3-*O*-glucoside, quercetin 3-*O*-galactoside, quercetin 3-*O*-rutinoside, quercetin 3-*O*-glucuronide, quercetin and gallic acid were purchased from Extrasynthese (Genay, France). Urolithin A 3-*O*-glucuronide and urolithin B 3-*O*-glucuronide were purchased from Toronto Research Chemicals (Toronto, ON, Canada). Epicatechin, ellagic acid, 2-hydroxybenzoic acid, 3-hydroxybenzoic acid, 4-hydroxybenzoic acid, 2,3-dihydroxy-benzoic acid, 2,5-dihydroxybenzoic acid, 3,4-dihydroxybenzoic acid, syringic acid, 4-hydroxy-benzaldehyde, 3,4-dihydroxybenzaldehyde, phloroglucinaldehyde, vanillin, *p*-coumaric acid, *m*-coumaric acid, *o*-coumaric acid, caffeic acid, sinapic acid, hippuric acid, 2-methylhippuric acid, 4-methylhippuric acid, 2-phenylacetic acid, 2-hydroxyphenylacetic acid, 3-hydroxyphenylacetic acid, 4-hydroxyphenylacetic acid, 4-methoxyphenylacetic acid, 3,4-dihydroxyphenylacetic acid, homovanillic acid, 3-(3-hydroxyphenyl)propionic acid, 3-(4-hydroxyphenyl)propionic acid, hydrocaffeic acid, hydroferulic acid and taxifolin were purchased from Sigma-Aldrich (St. Louis, MO, USA). Ferulic acid and vanillic acid were purchased from Honeywell (Charlotte, NC, USA). Methanol, acetone and acetonitrile were purchased from Thermo Fisher (Waltham, MA, USA). Formic acid and acetic acid were from Sigma-Aldrich. Ultrapure water was prepared with Millipore Direct-Q 3 Water Purification System (Burlington, MA, USA) and used throughout this study. All chemicals and reagents were of HPLC/MS grade.

### 4.7. Plasma and Urine Samples Processing and Analysis by UHPLC-QQQ

Blood samples were collected in vacutainers containing ethylenediaminetetraacetic acid (EDTA) and immediately placed on ice until centrifuged (within 30 min). After centrifugation at 453× *g* for 15 min at 4 °C, plasma was aliquoted into individual cryovials and stored at −80 °C until analysis. Spot urine samples were collected in urine collection cups, immediately placed on ice, and then aliquoted into individual cryovials and stored at −80 °C until analysis.

The plasma and urinary analysis of (poly)phenolic metabolites was performed using an UHPLC system couple with a triple quadrupole tandem mass spectrometer model 6460 (UHPLC-QQQ, Agilent Technologies, Santa Clara, CA, USA), operating in dynamic multiple reaction monitoring (dMRM) and negative/positive electrospray ionization (ESI) modes [2]. Plasma samples were extracted using solid phase extraction (SPE) C18 cartridges (3 mL et al., 200 mg; Agilent Technologies) and concentrated (8 times). Urine samples were filtered with a 0.2 μm polypropylene syringe filter (Whatman, Maidston, UK) and diluted (10 times) with staring mobile phase (5% acetonitrile containing 1% formic acid). Two internal standards (malvidin 3-O-glucoside and taxifolin) were added to each sample before SPE, with final concentrations 8 ppb and 64 ppb, respectively. Sample extracts (5 μL) were injected to a reversed-phase Poroshell 120 SB-C18 Stable Bond column (2.1 × 150 mm, 2.7 μm) equipped with a guard column (2.1 × 5 mm, 2.7 μm) for the separation of red raspberry (poly)phenols and their metabolites (except for phenolic acids) and a Pursuit 3 PFP column (2.0 × 150 mm, 3 μm) equipped with a guard column (2.0 × 2 mm, 3 μm) for the separation of phenolic acids and their derivatives. The columns and guard columns were purchased from Agilent Technologies. For the C18 column, the UHPLC mobile phase was 1% formic acid in water (A) and acetonitrile (B). The flow rate was 0.3 mL/min and a gradient consisted of 5% B at 0 min, 15% B at 10 min, 20% B at 12 min, 50% B at 20 min, 90% B at 23 min followed by 7 min post-run time for column re-equilibration. For the PFP column, the UHPLC mobile phase was 0.1% formic acid in water (A) and 0.1% formic acid in acetonitrile (B). The flow rate was 0.4 mL/min and a gradient consisted of 5% B at 0 min, 10% B at 3 min, 15% B at 7–9 min, 20% B at 10–11 min, 25% B at 12 min, 30% B at 13–14 min and 95% B at 15 min followed by 5-min post-run time for column re-equilibration. Metabolite identification was performed by multiple reaction monitoring (MRM) optimized with pure standards wherever possible and parent/daughter ion fragments obtained from an UHPLC coupled with quadruple time-of-flight (UHPLC-QTOF). Metabolites were confirmed on the basis of retention time (using authentic standards where possible) and three or more ion transitions. The UHPLC-QQQ method was validated for linearity, sensitivity, precision, recovery, and matrix effect (Table S4). Plasma (poly)phenol concentrations at 0, 1, 2, 3, 4 and 24 h were analyzed and expressed as nmol/L. Spot urine samples were pooled at time window 0 h, 1–4 h and 24 h for analysis. Urinary creatinine was measured using a RX Daytona automated clinical analyzer (Randox Laboratories, Crumlin, UK) with appropriate standards and quality controls. Urinary (poly)phenol concentrations were adjusted for creatinine concentration and expressed as nmol/μmol creatinine.

### 4.8. Data Analysis

The areas under the 24 h curve of (poly)phenolic metabolites (AUC_0–24h_) after each RRBtest drink was determined using the trapezoidal rule using Microsoft excel [19]. The time (T_max_) to achieve maximum plasma concentration (C_max_) were also evaluated. Randomization schedules, sample size estimates, and data analyses were performed using SAS 9.4 (SAS Institute, Inc., Cary, NC, USA).

Normal distribution of data was examined via the Shapiro-Wilk test. Non-normal distributed data were log10 transformed for analysis to normalize distributions. Data obtained at Baseline/Week 0 were used for subject characteristics. Repeated measures analysis of variance using mixed procedure was used for all endpoints assessing main effects of metabolic health status (PreDM-IR vs. Reference), Supplementation (baseline vs. RRB vs. RRB+FOS), Time (0, 1, 2, 3, 4, 24 h), and their 2-way interactions. Covariates (age, BMI, gender and race) were tested in all models and significant covariates were included in final analyses. These terms were removed from the model if not significant. When significant main effects and/or interactions were observed, post-hoc mean-separation testing was conducted using the Tukey-Kramer correction to adjust for multiple comparisons. Results are presented as means ± standard errors of the mean (SEM). Two-tailed *p* < 0.05 was considered significant.

## 5. Conclusions

The results from this study indicate the importance of gut microbiota in (poly)phenol metabolism, with microbial metabolites accounting for over 99% (poly)phenol metabolites in plasma and urine. Nutritional strategies incorporating RRB and FOS regularly in the diet increased microbial metabolites of (poly)phenols in the blood that were mostly lower in the PreDM-IR group compared to Reference group. These data suggest alterations in the capacity of the gut microbiome of individuals with PreDM-IR to metabolize and render bioavailable raspberry-derived (poly)phenols, but can be enhanced when consumed regularly for 4 weeks. The clinical importance of these findings is the subject for future work.

## Figures and Tables

**Table 1:** Subject demographic characteristics at baseline ^1^.

Variable ^2^	PreDM-IR (n = 25)	Reference (n = 10)	*p* Value
Age (years)	35 ± 2	31 ± 3	NS
Female: Male	11:14	7: 3	NS
CAU:AA:AS:HIS	8:6:8:3	3:2:3:2	NS
Fasting Glucose (mmol/L)	5.7 ± 0.1	5.1 ± 0.2	<0.0001
Fasting Insulin (pmol/L)	84 ± 9	34 ± 4	0.002
HOMA-IR	3.3 ± 0.4	1.0 ± 0.1	0.0005
BMI (kg/m^2^)	28 ± 1	22 ± 1	0.006
Weight (kg)	84 ± 4	64 ± 5	0.02

^1^ Mean ± standard error of the mean (SEM) for continuous variables. NS, non-significant. ^2^ CAU, Caucasian; AA, African American; AS, Asian; HIS, Hispanics and Latino; HOMA-IR, homeostasis model assessment of insulin resistance; BMI, body mass index.

**Table 2:** Red raspberry (poly)phenol contents in RRB-based test drink (RRBtest) and 4-week supplementations (mg/serving) ^1^.

RT	Compounds	MRM Transition	RRBtest Drink	4-Week Supplementations	
				Daily RRB Drink	Daily RRB+FOS Drink
6.5	Cyanidin 3,5-*O*-diglucoside	611^+^/287	0.8 ± 0.1	0.6 ± 0.0	0.5 ± 0.0
8.2	Cyanidin 3-*O*-sophoroside	611^+^/287	192.2 ± 2.2	61.0 ± 0.4	60.2 ± 1.1
9.1	Cyanidin 3-*O*-sambubioside	581^+^/287	10.1 ± 0.1	2.9 ± 0.0	2.4 ± 0.1
9.2	Cyanidin 3-*O*-glucoside	449^+^/287	27.4 ± 0.1	8.9 ± 0.0	9.9 ± 0.3
9.4	Pelargonidin 3-*O*-sophoroside	595^+^/271	6.3 ± 0.1	3.6 ± 0.1	3.4 ± 0.1
10.5	Pelargonidin 3-*O*-glucoside	433^+^/271	0.5 ± 0.0	0.3 ± 0.0	0.3 ± 0.0
	Total anthocyanins		236.8 ± 1.9	77.3 ± 0.3	76.7 ± 1.3
3.5	Pedunculagin isomer 1	783^−^/301	0.5 ± 0.0	1.2 ± 0.0	0.9 ± 0.0
5.2	Pedunculagin isomer 2	783^−^/301	0.3 ± 0.0	0.6 ± 0.0	0.4 ± 0.0
6.8	Sanguiin H-10 isomer 1	783^2−^/301	1.7 ± 0.1	0.7 ± 0.0	0.7 ± 0.0
6.8	Sanguiin H-6 minus gallic moiety isomer 1	858^2−^/301	1.0 ± 0.0	0.5 ± 0.0	0.5 ± 0.0
7.4	Corilagin	633^−^/301	0.3 ± 0.0	0.1 ± 0.0	0.1 ± 0.0
10.0	Sanguiin H-10 isomer 2	783^2−^/301	3.6 ± 0.1	1.6 ± 0.0	1.5 ± 0.1
11.1	Sanguiin H-6 minus gallic moiety isomer 2	858^2−^/301	0.3 ± 0.0	0.3 ± 0.0	0.2 ± 0.0
11.3	Sanguiin H-10 isomer 3	783^2−^/301	1.1 ± 0.1	0.2 ± 0.1	0.2 ± 0.0
11.9	Lambertianin C	1401^2−^/301	22.7 ± 0.6	8.0 ± 0.7	8.3 ± 0.3
12.3	Sanguiin H6	934^2−^/301	81.4 ± 1.7	25.2 ± 1.3	25.5 ± 0.5
13.1	Ellagic acid pentoside isomer 1	433^−^/301	1.7 ± 0.0	0.8 ± 0.0	0.7 ± 0.0
13.3	Ellagic acid pentoside isomer 2	433^−^/301	2.2 ± 0.1	0.7 ± 0.0	0.7 ± 0.0
13.6	Ellagic acid	301^−^/301	5.9 ± 0.1	2.0 ± 0.1	2.3 ± 0.2
14.9	Methyl ellagic acid pentoside 1	447^−^/301	0.6 ± 0.0	0.2 ± 0.0	0.2 ± 0.0
15.2	Methyl ellagic acid pentoside 2	447^−^/301	0.3 ± 0.0	0.1 ± 0.0	0.1 ± 0.0
15.2	Ellagic acid acetyl pentoside isomer 1	475^−^/301	0.3 ± 0.0	0.2 ± 0.0	0.2 ± 0.0
15.5	Ellagic acid acetyl pentoside isomer 2	475^−^/301	0.6 ± 0.0	0.2 ± 0.0	0.2 ± 0.0
	Total ellagic acid and ETs		124.5 ± 2.4	42.6 ± 1.7	42.7 ± 0.5
6.1	Procyanidin B EC/EC ^2^ dimer 1	577^−^/289	0.7 ± 0.1	0.2 ± 0.0	0.2 ± 0.0
6.7	Catechin	289^−^/125	0.6 ± 0.1	0.1 ± 0.0	0.2 ± 0.0
7.8	Procyanidin B EC/EC ^2^ dimer 2	577^−^/289	7.9 ± 0.6	1.8 ± 0.0	2.3 ± 0.1
8.3	Procyanidin B EC/EC ^2^ dimer 3	577^−^/289	0.4 ± 0.0	0.1 ± 0.0	0.2 ± 0.0
8.4	Proanthocyanidin EF/EC ^2^ dimer 1	561^−^/289	0.4 ± 0.0	0.1 ± 0.0	0.2 ± 0.0
9.4	Epicatechin	289^−^/125	9.0 ± 0.4	2.8 ± 0.1	2.6 ± 0.2
9.6	Proanthocyanidin EF/EC/EC ^2^ trimer	849^−^/289	0.6 ± 0.0	0.1 ± 0.0	0.2 ± 0.0
9.7	Proanthocyanidin EF/EC ^2^ dimer 2	561^−^/289	0.2 ± 0.0	0.1 ± 0.0	0.1 ± 0.0
10.4	Proanthocyanidin EF/EF/EC ^2^ trimer 1	833^−^/289	0.2 ± 0.0	0.1 ± 0.0	0.1 ± 0.0
12.1	Proanthocyanidin EF/EF/EC ^2^ trimer 2	833^−^/289	1.1 ± 0.1	0.3 ± 0.0	0.4 ± 0.0
	Total flavan-3-ols		21.1 ± 1.0	5.7 ± 0.2	6.5 ± 0.2
11.5	Quercetin 3-*O*-galactosylglucoside	625^−^/301	1.4 ± 0.1	0.3 ± 0.0	0.3 ± 0.0
11.8	Quercetin 3-*O*-sophoroside	625^−^/301	0.6 ± 0.0	0.3 ± 0.0	0.3 ± 0.0
12.9	Quercetin 3-*O*-galactosylrhamnoside	609^−^/301	0.1 ± 0.0	0.1 ± 0.0	0.1 ± 0.0
13.9	Quercetin 3-*O*-galactoside	463^−^/301	0.4 ± 0.0	0.1 ± 0.0	0.1 ± 0.0
14.1	Quercetin 3-*O*-glucosde	463^−^/301	0.3 ± 0.0	0.2 ± 0.0	0.2 ± 0.0
14.2	Quercetin 3-*O*-glucuronide	477^−^/301	0.4 ± 0.0	0.5 ± 0.0	0.5 ± 0.0
	Total flavonols		3.1 ± 0.1	1.5 ± 0.0	1.5 ± 0.0
2.4	Gallic acid	169^−^/125	0.7 ± 0.1	0.1 ± 0.0	0.2 + 0.0
4.1	3,4-Dihydroxybenzoic acid	153^−^/109	0.1 ± 0.0	0.0 ± 0.0	0.1 ± 0.0
5.3	Caffeoyl hexoside isomer 1	341^−^/179	1.3 ± 0.0	1.9 ± 0.0	1.7 ± 0.0
6.7	Caffeoyl hexoside isomer 2	341^−^/179	0.4 ± 0.0	0.3 ± 0.0	0.3 ± 0.0
7.2	*p*-Coumaryl hexoside	325^−^/145	0.4 ± 0.0	0.3 ± 0.0	0.2 ± 0.0
	Total phenolic acids		2.9 ± 0.1	2.6 ± 0.0	2.4 ± 0.0
	Total (poly)phenols		388.4 ± 3.3	129.7 ± 1.9	129.8 ± 0.5

^1^ Analysis was conducted in triplicate, data are presented as mean ± standard deviation (SD); ^2^ EC, epicatechin/catechin; EF, epifisetinidol/fisetinidol.

## Supplementary Materials

The following are available online, Table S1: Effect of 4-week RRB and RRB+FOS supplementations on plasma and urinary (poly)phenolic metabolites profiling tested by AUC_0–24h_ post the RRBtest drink, Table S2: Nutrient composition of RRB-based test drink (RRBtest) and 4-week supplementary drinks, Table S3: Dietary assessment during 4-week RRB and RRB+FOS supplementations, Table S4: Method validation for linearity, limit of detection (LOD) and limit of quantification (LOQ), precision (within-run and between-run), recovery and matrix effect using standards dissolved in starting mobile phase and in biological samples (plasma and urine).
