# Supplementary material for: Plasma and Urinary (Poly)phenolic Profiles after 4-Week Red Raspberry (Rubus idaeus L.) Intake with or without Fructo-Oligosaccharide Supplementation
Source: Molecules. 2020 Oct 17;25(20):4777. doi: 10.3390/molecules25204777 (PMC7594073; doi:10.3390/molecules25204777)
Supplement: Supplementary file 1 [file molecules-25-04777-s001.pdf]

Supplementary Materials

Table S1 Effect of 4-week RRB and RRB+FOS supplementations on plasma and urinary (poly)phenolic metabolites profiling tested by $AUC_{0\text{-}24h}$ post the RRBtest drink*.

(poly)phenolic Metabolites	MRM transitions		Baseline	4-week RRB	4-week RRB+FOS	Baseline vs RRB *p* value	Baseline vs. RRB+FOS *p* value	RRB vs. RRB+FOS *p* value
**Anthocyanin derivatives**		Plasma	438 ± 39	360 ± 41	394 ± 36	0.0026	0.0248	NS
		Urine	3.5 ± 0.4	2.7 ± 0.2	2.5 ± 0.3	NS	NS	NS
Cyanidin 3-O-sophoroside#	611/287	Plasma	273 ± 25	217 ± 30	234 ± 24	0.0014	0.0055	NS
		Urine	2.4 ± 0.3	1.8 ± 0.2	1.7 ± 0.2	NS	NS	NS
Cyanidin 3-O-glucoside	449/287	Plasma	31 ± 2	36 ± 6	34 ± 3	NS	NS	NS
		Urine	0.3 ± 0	0.3 ± 0	0.3 ± 0	NS	NS	NS
Cyanidin 3-O-sambubioside#	581/287	Plasma	15 ± 2	13 ± 2	13 ± 2	NS	NS	NS
		Urine	0.1 ± 0.01	0.07 ± 0.01	0.07 ± 0.01	NS	NS	NS
Pelargonidin 3-O-sophoroside#	595/271	Plasma	7 ± 1	5 ± 1	6 ± 1	0.0001	0.0005	NS
		Urine	0.09 ± 0.01	0.06 ± 0.01	0.06 ± 0.01	NS	NS	NS
Pelargonidin 3-O-glucoside	433/271	Plasma	2 ± 0.1	2 ± 0.1	2 ± 0.1	NS	NS	NS
		Urine	0.002 ± 0.001	0.002 ± 0.001	0.002 ± 0.001	NS	NS	NS
Methyl cyanidin glucuronide#	477/301	Plasma	57 ± 12	48 ± 10	57 ± 10	NS	NS	NS
		Urine	0.2 ± 0.1	0.2 ± 0.0	0.2 ± 0.0	NS	NS	NS
Cyanidin glucuronide#	463/287	Plasma	5 ± 1	3 ± 1	4 ± 1	NS	NS	NS
		Urine	0.05 ± 0.01	0.03 ± 0.01	0.03 ± 0.01	NS	NS	NS
Cyanidin diglucuronide#	639/287	Plasma	3 ± 1	2 ± 1	3 ± 0	NS	NS	NS
		Urine	-	-	-	-	-	-
Methyl cyanidin 3-O-sophoroside#	625/301	Plasma	18 ± 2	14 ± 1	16 ± 2	NS	NS	NS
		Urine	0.12 ± 0.02	0.08 ± 0.01	0.08 ± 0.01	NS	NS	NS
Methyl cyanidin 3-O-glucoside	463/301	Plasma	3 ± 0.2	3 ± 0.3	3 ± 0.2	NS	NS	NS
		Urine	0.06 ± 0.01	0.06 ± 0.01	0.06 ± 0.01	NS	NS	NS
Methyl cyanidin 3-O-sambubioside#	595/301	Plasma	0.3 ± 0.1	0.2 ± 0.1	0.3 ± 0.1	NS	NS	NS
		Urine	-	-	-	-	-	-
Methyl cyanidin 3-O-sophoroside glucuronide#	801/301	Plasma	0.3 ± 0.1	0.3 ± 0.1	0.4 ± 0.1	NS	NS	NS
		Urine	-	-	-	-	-	-
Pelargonidin glucuronide#	447/271	Plasma	26 ± 5	17 ± 3	23 ± 3	0.0018	NS	0.0103
		Urine	0.13 ± 0.03	0.08 ± 0.02	0.1 ± 0.02	NS	NS	NS
**Urolithin derivatives**		Plasma	429 ± 72	1044 ± 288	1110 ± 403	0.0001	0.0001	NS
		Urine	23 ± 4	51 ± 10	66 ± 34	0.0001	0.0001	NS
Urolithin A#	227/198	Plasma	-	-	-	-	-	-
		Urine	0.2 ± 0.2	0 ± 0	2.4 ± 2.3	NS	0.0099	0.0021
Urolithin A 3-O-glucuronide	403/227	Plasma	311 ± 63	569 ± 125	887 ± 406	0.0001	0.0001	NS
		Urine	14.7 ± 2.6	26.7 ± 5.1	46.8 ± 27.8	0.0001	0.0001	NS
Urolithin A glucuronide#	403/227	Plasma	91 ± 38	133 ± 45	261 ± 116	0.0001	0.0014	NS
		Urine	7.7 ± 3.3	12.6 ± 4.5	13.7 ± 4.7	0.0014	0.0017	NS

Isourolithin A glucuronide#	403/227	Plasma	129 ± 85	112 ± 69	91 ± 64	NS	NS	NS
		Urine	1.9 ± 1.7	0.2 ± 0.1	1 ± 0.6	NS	NS	NS
Urolithin A sulfoglucuronide#	483/227	Plasma	-	-	-	-	-	-
		Urine	2.3 ± 0.5	4.6 ± 1	11.9 ± 8.2	0.0001	0.0001	NS
Urolithin B 3-O-glucuronide	387/211	Plasma	130 ± 64	1376 ± 762	305 ± 97	0.0001	0.0001	0.0001
		Urine	5.3 ± 1.8	44.4 ± 20.6	9 ± 2.3	0.0028	NS	0.0078
Urolithin B sulfoglucuronide#	467/211	Plasma	-	-	-	-	-	-
		Urine	1.0 ± 0.4	3.9 ± 1.3	3.4 ± 1.1	0.0004	0.0009	NS
**Phenyl-γ-valerolactone derivatives**		Plasma	2224 ± 495	2853 ± 682	2536 ± 502	0.0196	0.001	NS
		Urine	768 ± 202	1149 ± 256	898 ± 254	0.0001	0.0001	NS
5-(3′,4′-dihydroxyphenyl)-γ-valerolactone glucuronide isomer 1#	383/207	Plasma	526 ± 130	743 ± 186	627 ± 146	0.0011	0.0383	NS
		Urine	5 ± 1	9 ± 2	7 ± 2	0.0401	0.0124	NS
5-(3′,4′-dihydroxyphenyl)-γ-valerolactone glucuronide isomer 2#	383/207	Plasma	1441 ± 354	1805 ± 466	1612 ± 363	0.0014	0.0001	NS
		Urine	16 ± 4	29 ± 8	19 ± 4	0.0118	NS	NS
5-(3′,4′-dihydroxyphenyl)-γ-valerolactone sulfate isomer 1#	287/207	Plasma	-	-	-	-	-	-
		Urine	13 ± 8	55 ± 31	10 ± 3	0.0222	NS	NS
5-(3′,4′-dihydroxyphenyl)-γ-valerolactone sulfate isomer 2#	287/207	Plasma	-	-	-	-	-	-
		Urine	520 ± 186	868 ± 243	644 ± 242	0.0001	0.0001	NS
5-(4′-hydroxyphenyl)-γ-valerolactone glucuronide isomer 1#	367/191	Plasma	12 ± 7	48 ± 30	10 ± 7	0.0179	NS	0.0073
		Urine	0.5 ± 0.1	0.8 ± 0.4	0.8 ± 0.2	NS	NS	NS
5-(4′-hydroxyphenyl)-γ-valerolactone glucuronide isomer 2#	367/191	Plasma	112 ± 53	74 ± 38	104 ± 48	NS	NS	NS
		Urine	2 ± 1	1 ± 1	2 ± 1	NS	NS	NS
5-(4′-hydroxyphenyl)-γ-valerolactone sulfate#	271/191	Plasma	-	-	-	-	-	-
		Urine	153 ± 61	118 ± 41	160 ± 59	NS	NS	NS
5-(3′,4′, 5'-trihydroxyphenyl)-γ-valerolactone glucuronide#	399/223	Plasma	134 ± 29	183 ± 49	183 ± 38	NS	NS	NS
		Urine	3 ± 1	4 ± 1	3 ± 1	NS	NS	NS
5-(3′,4′, 5'-dihydroxyphenyl)-γ-valerolactone sulfate#	303/223	Plasma	-	-	-	-	-	-
		Urine	56 ± 12	64 ± 24	53 ± 12	NS	NS	NS
**Phenolic acid derivatives**		Plasma	444830 ± 51957	551588 ± 87408	582308 ± 74446	0.0053	0.0024	NS
		Urine	45907 ± 3756	38198 ± 3585	39774 ± 2930	NS	NS	NS
Benzaldehyde derivatives		Plasma	3505 ± 172	3334 ± 137	3568 ± 158	NS	NS	NS
		Urine	28 ± 3	26 ± 3	27 ± 3	NS	NS	NS
4-Hydroxybenzaldehyde	121/92	Plasma	2974 ± 116	2628 ± 123	3083 ± 137	0.0439	NS	0.0176
		Urine	3 ± 1	3 ± 1	2 ± 0	NS	NS	NS
Hydroxybenzaldehyde sulfate#	201/121	Plasma	369 ± 97	251 ± 41	311 ± 44	0.0232	NS	0.0255
		Urine	11 ± 1	9 ± 1	9 ± 1	0.0127	NS	NS
3,4-Dihydroxybenzaldehyde	137/108	Plasma	16 ± 4	16 ± 4	15 ± 3	NS	NS	NS
		Urine	0.6 ± 0.5	0.4 ± 0.2	0.8 ± 0.4	NS	NS	NS

Dihydroxybenzaldehyde sulfate isomer 1#	217/108	Plasma	6 ± 4	3 ± 3	5 ± 4	NS	NS	NS
		Urine	3 ± 1	3 ± 1	2 ± 1	NS	NS	NS
Dihydroxybenzaldehyde sulfate isomer 2#	217/108	Plasma	9 ± 4	7 ± 4	14 ± 9	NS	NS	NS
		Urine	6 ± 2	5 ± 2	5 ± 2	NS	NS	NS
Phloroglucinaldehyde	153/151	Plasma	91 ± 11	73 ± 11	89 ± 12	0.015	NS	0.0278
		Urine	0.1 ± 0.03	0.03 ± 0.02	0.02 ± 0.01	NS	NS	NS
Phloroglucinaldehyde sulfate#	233/151	Plasma	18 ± 7	33 ± 16	27 ± 6	NS	NS	NS
		Urine	1.2 ± 0.2	1.6 ± 0.3	1.4 ± 0.2	NS	NS	NS
Vanillin	151/136	Plasma	180 ± 13	186 ± 16	174 ± 13	NS	NS	NS
		Urine	0.3 ± 0.1	0.2 ± 0.1	0.3 ± 0.1	NS	NS	NS
Vanillin sulfate isomer 1#	231/136	Plasma	11 ± 9	20 ± 14	10 ± 9	NS	NS	NS
		Urine	20 ± 2	22 ± 3	19 ± 2	NS	NS	NS
Vanillin sulfate isomer 2#	231/136	Plasma	803 ± 623	714 ± 639	551 ± 309	NS	NS	NS
		Urine	19 ± 5	24 ± 9	18 ± 5	NS	NS	NS
Cinnamic acid derivatives		Plasma	2199 ± 196	2479 ± 270	2761 ± 231	0.0203	0.0015	NS
		Urine	836 ± 74	901 ± 107	878 ± 84	NS	NS	NS
o-Coumaric acid	163/119	Plasma	5 ± 2	11 ± 4	6 ± 2	NS	NS	NS
		Urine	0.01 ± 0.00	0.04 ± 0.02	0.1 ± 0.04	NS	NS	NS
m-Coumaric acid	163/119	Plasma	11 ± 6	11 ± 6	11 ± 5	NS	NS	NS
		Urine	0.5 ± 0.3	0.3 ± 0.1	0.5 ± 0.2	NS	NS	NS
p-Coumaric acid	163/119	Plasma	29 ± 5	30 ± 6	30 ± 5	NS	NS	NS
		Urine	0.2 ± 0.1	0.3 ± 0.1	0.2 ± 0.1	NS	NS	NS
Coumaric acid glucuronide isomer 1#	339/163	Plasma	2 ± 2	0.4 ± 0.4	2 ± 1	NS	NS	NS
		Urine	-	-	-	-	-	-
Coumaric acid glucuronide isomer 2#	339/163	Plasma	5 ± 2	3 ± 1	4 ± 2	NS	NS	NS
		Urine	0.5 ± 0.2	0.3 ± 0.1	0.3 ± 0.1	NS	NS	NS
Coumaric acid sulfate isomer 1#	243/163	Plasma	-	-	1 ± 1	NS	NS	NS
		Urine	5 ± 1	7 ± 3	4 ± 1	NS	NS	NS
Coumaric acid sulfate isomer 2#	243/163	Plasma	29 ± 10	27 ± 11	35 ± 11	NS	NS	NS
		Urine	16 ± 3	16 ± 3	14 ± 3	NS	NS	NS
Coumaric acid sulfate isomer 3#	243/163	Plasma	18 ± 5	15 ± 8	29 ± 13	NS	NS	NS
		Urine	22 ± 3	19 ± 3	19 ± 2	NS	NS	NS
Coumaric acid sulfate isomer 4#	243/163	Plasma	2 ± 1	7 ± 3	10 ± 4	NS	NS	NS
		Urine	4 ± 2	3 ± 1	3 ± 1	NS	NS	NS
Caffeic acid	179/135	Plasma	437 ± 50	475 ± 62	468 ± 53	0.0471	NS	NS
		Urine	11 ± 1	12 ± 2	10 ± 1	NS	NS	NS
Caffeic acid glucuronide isomer 1#	355/179	Plasma	5 ± 5	3 ± 3	3 ± 3	NS	NS	NS
		Urine	0.1 ± 0.1	0.1 ± 0.1	0.3 ± 0.1	NS	NS	NS
Caffeic acid glucuronide isomer 2#	355/179	Plasma	7 ± 7	-	4 ± 4	NS	NS	NS
		Urine	0.1 ± 0.1	0.1 ± 0	0.2 ± 0.1	NS	NS	NS
Caffeic acid sulfate isomer 1#	259/179	Plasma	11 ± 6	24 ± 24	18 ± 10	NS	NS	NS
		Urine	4 ± 1	4 ± 1	6 ± 2	NS	NS	NS
Caffeic acid sulfate isomer 2#	259/179	Plasma	48 ± 19	31 ± 18	62 ± 17	NS	NS	NS

		Urine	66 ± 7	77 ± 7	73 ± 6	NS	NS	NS
Caffeic acid sulfate isomer 3#	259/179	Plasma	64 ± 29	69 ± 32	123 ± 45	NS	NS	NS
		Urine	50 ± 6	35 ± 5	37 ± 5	NS	NS	NS
Ferulic acid	193/134	Plasma	7 ± 3	10 ± 3	8 ± 2	NS	NS	NS
		Urine	-	-	-	-	-	-
Isoferulic acid#	193/134	Plasma	103 ± 14	112 ± 17	123 ± 16	NS	0.0355	NS
		Urine	6 ± 1	7 ± 1	6 ± 1	NS	NS	NS
Ferulic acid hydrate isomer 1#	211/136	Plasma	46 ± 14	36 ± 13	74 ± 35	NS	0.0339	NS
		Urine	3 ± 1	3 ± 1	6 ± 4	NS	0.0038	NS
Ferulic acid hydrate isomer 2#	211/136	Plasma	78 ± 12	94 ± 17	110 ± 17	0.0449	0.0005	NS
		Urine	6 ± 1	7 ± 2	7 ± 1	NS	NS	NS
Ferulic acid glucuronide isomer 1#	369/193	Plasma	567 ± 59	696 ± 100	666 ± 67	0.005	0.0475	NS
		Urine	3 ± 0	4 ± 1	4 ± 1	NS	NS	NS
Ferulic acid glucuronide isomer 2#	369/193	Plasma	294 ± 53	240 ± 38	364 ± 55	NS	NS	NS
		Urine	3 ± 0	3 ± 1	4 ± 1	NS	NS	NS
Ferulic acid sulfate#	273/193	Plasma	5 ± 4	7 ± 7	108 ± 95	NS	NS	NS
		Urine	4222 ± 367	4988 ± 698	4781 ± 522	NS	NS	NS
Sinapic acid glucuronide#	399/233	Plasma	412 ± 66	569 ± 146	491 ± 78	NS	NS	NS
		Urine	6 ± 1	8 ± 2	6 ± 1	NS	NS	NS
Sinapic acid sulfate#	303/233	Plasma	-	-	-	-	-	-
		Urine	160 ± 36	139 ± 17	146 ± 21	NS	NS	NS
Phenylpropionic acid derivatives		Plasma	110360 ± 14315	161139 ± 30800	166157 ± 23449	0.0001	0.0001	NS
		Urine	10745 ± 1659	15058 ± 3176	12213 ± 2265	0.0416	NS	NS
3-(3-Hydroxyphenyl)propionic acid	179/164	Plasma	3225 ± 918	2998 ± 878	3400 ± 671	NS	NS	0.001
		Urine	10 ± 7	8 ± 6	7 ± 4	NS	NS	NS
3-(4-Hydroxyphenyl)propionic acid	179/164	Plasma	49032 ± 1929	60455 ± 3664	64543 ± 3294	0.0001	0.0001	NS
		Urine	7744 ± 1628	11239 ± 3091	8747 ± 2181	0.0439	NS	NS
Hydroxyphenylpropionic acid sulfate#	245/121	Plasma	25 ± 17	9 ± 9	31 ± 18	NS	NS	NS
		Urine	48 ± 7	52 ± 8	50 ± 6	NS	NS	NS
Methoxyphenylpropionic acid#	179/164	Plasma	5236 ± 1796	7248 ± 1757	7414 ± 1652	0.0001	0.0001	NS
		Urine	1 ± 1	2 ± 1	2 ± 1	NS	NS	NS
Methoxyphenylpropionic acid glucuronide isomer 1#	355/179	Plasma	54 ± 30	50 ± 21	68 ± 27	NS	0.0027	NS
		Urine	3 ± 1	4 ± 1	4 ± 1	0.0089	0.0103	NS
Methoxyphenylpropionic acid glucuronide isomer 2#	355/179	Plasma	210 ± 76	296 ± 71	373 ± 116	0.0001	0.0001	NS
		Urine	2 ± 1	7 ± 2	5 ± 1	0.0007	NS	NS
Methoxyphenylpropionic acid sulfate isomer 1#	259/179	Plasma	268 ± 36	267 ± 46	237 ± 35	NS	NS	NS
		Urine	92 ± 17	100 ± 18	115 ± 26	NS	NS	NS
Methoxyphenylpropionic acid sulfate isomer 2#	259/179	Plasma	316 ± 116	161 ± 30	245 ± 48	NS	NS	NS
		Urine	55 ± 19	74 ± 33	24 ± 8	NS	NS	NS
Dihydrocaffeic acid	181/137	Plasma	24 ± 24	37 ± 28	55 ± 37	NS	NS	NS
		Urine	32 ± 5	34 ± 5	37 ± 5	NS	NS	NS
Dihydrocaffeic acid grucuronide#	357/137	Plasma	789 ± 354	657 ± 186	549 ± 270	NS	NS	NS
		Urine	14 ± 5	21 ± 7	13 ± 5	NS	NS	NS

Dihydrocaffeic acid sulfate#	261/137	Plasma	-	-	-	-	-	-
		Urine	88 ± 26	134 ± 47	129 ± 36	NS	NS	NS
Dihydroferulic acid	195/136	Plasma	230 ± 70	220 ± 72	323 ± 114	NS	NS	NS
		Urine	3 ± 2	3 ± 2	10 ± 8	NS	NS	NS
Dihydroisoferulic acid#	195/136	Plasma	39556 ± 11076	51004 ± 10521	57878 ± 14024	0.0026	0.0337	NS
		Urine	406 ± 125	676 ± 176	570 ± 123	NS	NS	NS
Hydroxymethoxyphenylpropionic acid glucuronide isomer 1#	371/195	Plasma	1917 ± 606	2606 ± 621	3453 ± 1213	0.0001	0.0024	NS
		Urine	5 ± 2	7 ± 3	8 ± 3	NS	NS	NS
Hydroxymethoxyphenylpropionic acid glucuronide isomer 2#	371/195	Plasma	4354 ± 768	32112 ± 28167	21685 ± 17550	NS	NS	NS
		Urine	3 ± 2	104 ± 101	36 ± 33	NS	NS	NS
Hydroxymethoxyphenylpropionic acid glucuronide isomer 3#	371/195	Plasma	913 ± 216	809 ± 192	1234 ± 291	NS	0.0069	0.0224
		Urine	12 ± 3	17 ± 6	20 ± 9	NS	NS	NS
Hydroxymethoxyphenylpropionic acid glucuronide isomer 4#	371/195	Plasma	899 ± 201	624 ± 162	1236 ± 314	NS	NS	NS
		Urine	46 ± 9	54 ± 13	63 ± 16	NS	NS	NS
Hydroxymethoxyphenylpropionic acid sulfate isomer 1#	275/195	Plasma	1499 ± 759	587 ± 400	2181 ± 915	NS	NS	NS
		Urine	1131 ± 195	1319 ± 273	1231 ± 200	NS	NS	NS
Hydroxymethoxyphenylpropionic acid sulfate isomer 2#	275/195	Plasma	1640 ± 1051	761 ± 343	1097 ± 474	NS	NS	NS
		Urine	1051 ± 259	1202 ± 358	1137 ± 272	NS	NS	NS
Phenylacetic acid derivatives		Plasma	131264 ± 36541	115419 ± 33273	155156 ± 34731	NS	NS	NS
		Urine	6818 ± 662	7430 ± 968	6246 ± 545	NS	NS	NS
Phenylacetic acid	135/91	Plasma	4430 ± 1283	4084 ± 1210	3630 ± 1098	NS	NS	NS
		Urine	-	-	-	-	-	-
Phenylacetic acid sulfate#	215/135	Plasma	110588 ± 36365	94969 ± 33318	131319 ± 34502	NS	NS	NS
		Urine	4568 ± 472	4740 ± 531	4027 ± 381	NS	NS	NS
2-Hydroxyphenylacetic acid	151/107	Plasma	905 ± 57	864 ± 61	949 ± 66	NS	NS	0.0133
		Urine	3 ± 0	2 ± 0	3 ± 0	NS	NS	NS
3-Hydroxyphenylacetic acid	151/107	Plasma	1608 ± 334	1321 ± 328	1026 ± 214	0.0075	0.0001	NS
		Urine	67 ± 13	67 ± 19	45 ± 9	NS	0.0415	NS
4-Hydroxyphenylacetic acid	151/107	Plasma	6860 ± 1572	7748 ± 2161	8392 ± 1572	NS	NS	NS
		Urine	515 ± 54	503 ± 74	513 ± 62	NS	NS	NS
Hydroxyphenylacetic acid glucuronide#	327/107	Plasma	48 ± 36	79 ± 48	16 ± 16	NS	NS	NS
		Urine	-	-	-	-	-	-
Hydroxyphenylacetic acid sulfate#	231/107	Plasma	249 ± 46	245 ± 44	226 ± 42	NS	NS	NS
		Urine	10 ± 1	9 ± 1	9 ± 1	NS	NS	NS
4-methoxyphenylacetic acid	165/106	Plasma	2933 ± 823	2758 ± 780	3096 ± 599	NS	NS	0.0055
		Urine	9 ± 7	8 ± 6	7 ± 4	NS	NS	NS
3, 4-dihydroxyphenylacetic acid	167/123	Plasma	114 ± 23	109 ± 27	113 ± 37	NS	NS	NS
		Urine	33 ± 8	42 ± 10	32 ± 7	NS	NS	NS
Dihydroxyphenylacetic acid isomer 1#	167/123	Plasma	633 ± 112	585 ± 98	687 ± 122	NS	NS	NS
		Urine	74 ± 6	73 ± 10	63 ± 6	NS	NS	NS
Dihydroxyphenylacetic acid isomer 2#	167/123	Plasma	33 ± 10	76 ± 34	66 ± 28	0.0342	0.0254	NS

		Urine	-	0.6 ± 0.4	0.2 ± 0.1	NS	NS	NS
Dihydroxyphenylacetic acid sulfate#	247/123	Plasma	2034 ± 196	1712 ± 215	1989 ± 184	NS	NS	NS
		Urine	1042 ± 185	1418 ± 353	988 ± 164	NS	NS	NS
Homovanillic acid	181/137	Plasma	683 ± 237	780 ± 323	645 ± 237	NS	NS	NS
		Urine	79 ± 22	64 ± 17	55 ± 13	NS	NS	NS
Homovanillic acid sulfate isomer 1#	261/122	Plasma	23 ± 22	89 ± 61	1466 ± 1466	NS	NS	NS
		Urine	234 ± 33	271 ± 62	261 ± 44	NS	NS	NS
Homovanillic acid sulfate isomer 2#	261/122	Plasma	-	-	-	-	-	-
		Urine	128 ± 32	169 ± 82	120 ± 33	NS	NS	NS
Dimethoxyhydroxyphenylacetic acid sulfate 1#	291/211	Plasma	-	-	-	-	-	-
		Urine	25 ± 5	33 ± 6	31 ± 5	NS	NS	NS
Dimethoxyhydroxyphenylacetic acid sulfate 2#	291/211	Plasma	-	-	-	-	-	-
		Urine	29 ± 7	31 ± 10	55 ± 15	NS	NS	NS
Benzoic acid derivatives		Plasma	108810 ± 13833	116906 ± 20175	122176 ± 15723	NS	0.0388	NS
		Urine	5919 ± 1288	5888 ± 861	4621 ± 536	NS	NS	NS
2-Hydroxybenzoic acid	137/93	Plasma	3691 ± 546	17555 ± 14399	12199 ± 8532	NS	0.0302	NS
		Urine	0.4 ± 0.2	10.2 ± 10	4.1 ± 3.8	NS	NS	NS
3-Hydroxybenzoic acid	137/93	Plasma	447 ± 151	370 ± 109	304 ± 75	NS	NS	NS
		Urine	0.4 ± 0.2	3.9 ± 3.3	0.5 ± 0.3	NS	NS	NS
4-Hydroxybenzoic acid	137/93	Plasma	976 ± 192	1313 ± 455	1249 ± 295	0.0498	0.0424	NS
		Urine	63 ± 8	62 ± 12	58 ± 9	NS	NS	NS
Hydroxybenzoic acid sulfate#	217/93	Plasma	603 ± 160	1148 ± 695	1240 ± 726	NS	NS	NS
		Urine	85 ± 9	93 ± 15	76 ± 10	NS	NS	NS
2,3-Dihydroxybenzoic acid	153/109	Plasma	63 ± 8	75 ± 16	61 ± 9	NS	NS	NS
		Urine	0.2 ± 0	0.2 ± 0.1	0.1 ± 0	NS	NS	NS
2,5-Dihydroxybenzoic acid	153/109	Plasma	795 ± 137	1061 ± 260	855 ± 172	NS	NS	NS
		Urine	7 ± 1	12 ± 3	7 ± 1	NS	NS	NS
3,4-Dihydroxybenzoic acid	153/109	Plasma	150 ± 22	170 ± 25	144 ± 16	0.0062	NS	0.0002
		Urine	10 ± 2	11 ± 2	10 ± 2	NS	NS	NS
Dihydroxybenzoic acid sulfate#	233/109	Plasma	2655 ± 319	2720 ± 328	2966 ± 342	0.0082	0.0001	NS
		Urine	104 ± 18	108 ± 15	79 ± 8	NS	NS	NS
Vanillic acid	167/152	Plasma	308 ± 165	252 ± 75	159 ± 41	NS	NS	NS
		Urine	-	-	-	-	-	-
Vanillic acid glucuronide isomer 1#	343/167	Plasma	3502 ± 868	4830 ± 1772	3236 ± 416	NS	NS	NS
		Urine	52 ± 29	93 ± 60	23 ± 4	NS	NS	NS
Vanillic acid glucuronide isomer 2#	343/167	Plasma	13626 ± 4253	12476 ± 2934	11374 ± 1578	NS	NS	NS
		Urine	91 ± 38	114 ± 57	58 ± 8	NS	NS	NS
Vanillic acid sulfate isomer 1#	247/167	Plasma	60352 ± 5485	54160 ± 5930	66115 ± 9906	NS	0.0492	0.0005
		Urine	3616 ± 591	3707 ± 599	2908 ± 437	NS	NS	NS
Vanillic acid sulfate isomer 2#	247/167	Plasma	18639 ± 5211	16276 ± 3529	18401 ± 3255	NS	NS	0.0243
		Urine	1857 ± 829	1592 ± 303	1331 ± 151	NS	NS	NS
Hydroxymethylbenzoate#	167/108	Plasma	2045 ± 198	2013 ± 260	2118 ± 210	NS	NS	NS
		Urine	2 ± 0	2 ± 1	2 ± 0	NS	NS	NS

Hydroxymethylbenzoate sulfate#	247/108	Plasma	-	-	-	-	-	-
		Urine	7 ± 4	9 ± 3	6 ± 2	NS	NS	NS
Syringic acid glucuronide#	373/197	Plasma	955 ± 69	945 ± 67	1032 ± 90	NS	NS	NS
		Urine	22 ± 2	22 ± 2	25 ± 4	NS	NS	NS
Hippuric acid derivatives		Plasma	87692 ± 7800	151391 ± 60182	131753 ± 37606	0.0415	0.0319	NS
		Urine	17753 ± 2180	20990 ± 3111	17171 ± 1748	NS	NS	NS
Hippuric acid	178/134	Plasma	68247 ± 6772	73493 ± 7769	73264 ± 7585	NS	NS	NS
		Urine	15097 ± 1967	16771 ± 2397	13813 ± 1387	NS	NS	NS
Hippuric acid glucuronide#	354/178	Plasma	836 ± 206	776 ± 138	859 ± 270	NS	NS	NS
		Urine	29 ± 10	23 ± 4	24 ± 6	NS	NS	NS
Hippuric acid sulfate isomer 1#	258/178	Plasma	776 ± 282	554 ± 220	623 ± 227	NS	NS	NS
		Urine	120 ± 41	177 ± 63	78 ± 21	NS	NS	NS
Hippuric acid sulfate isomer 2#	258/178	Plasma	388 ± 129	790 ± 471	592 ± 232	NS	NS	NS
		Urine	61 ± 16	86 ± 36	55 ± 21	NS	NS	NS
Hydroxyhippuric acid isomer 1#	194/150	Plasma	3433 ± 790	3498 ± 677	3708 ± 594	NS	NS	NS
		Urine	607 ± 141	604 ± 124	597 ± 87	NS	NS	NS
Hydroxyhippuric acid isomer 2#	194/150	Plasma	4617 ± 778	19737 ± 15167	14586 ± 9754	0.0487	0.0742	NS
		Urine	533 ± 97	1733 ± 1248	1142 ± 672	NS	NS	NS
Hydroxyhippuric acid glucuronide#	370/194	Plasma	6181 ± 1103	49906 ± 44143	33303 ± 27358	0.0043	0.0001	NS
		Urine	35 ± 8	220 ± 194	191 ± 163	NS	NS	NS
Hydroxyhippuric acid sulfate#	274/194	Plasma	3213 ± 512	2638 ± 390	3138 ± 467	NS	NS	NS
		Urine	1010 ± 145	1076 ± 133	973 ± 102	NS	NS	NS

*Plasma $AUC_{0\text{-}24h}$ was expressed as nmol/L h; Urine $AUC_{0\text{-}24h}$ was expressed as nmol h/μmol creatinine.

#These compounds were analyzed using parent compounds or structurally/chemically similar compounds.

Table S2 Nutrient composition of RRB-based test drink (RRBtest) and 4-week supplementary drinks[1]

Item name	RRBtest drink[2]	4-week Supplementations	
		Daily RRB drink[3]	Daily RRB+FOS drink[3]
Quantity (g)	414	98	106
Energy	361	45	77
Protein (g)	2.1	0.8	0.8
Fat (g)	1.4	0.6	0.6
Carbohydrate (g)	85	9	17
Fiber (g)	8	3	11
RRB fiber (g)	8	3	3
FOS (g)	0	0	8
Sugar (g)	75	5	5
RRB sugar	10	5	5
Added sugar	65	0	0
Total polyphenols (mg)	388	130	130
ACN (mg)	237	77	77
ET/EA (mg)	125	43	43

[1]Protein, carbohydrate, fiber and sugar content derived from USDA Food Composition Databases. Total polyphenols, anthocyanin (ACN) and ellagitannin/ellagic acid (ET/EA) contents were quantified using the method described in Zhang et al. 2018.)
[2]Containing single variety of 250 g IQF RRB (2 cups fresh weight equivalence).
[3]Containing multiple variety of 50 g IQF RRB and 8 g RRB freeze-dried powder (1 cup fresh weight equivalence), consumed one time per day.

Table S3 Dietary assessment during 4-week RRB and RRB+FOS supplementations*

Nutrient and food group	PreDM-IR		Reference	
	4-week RRB	4-week RRB+FOS	4-week RRB	4-week RRB+FOS
Energy (kcal	1964 ± 95	1934 ± 89	2194 ± 163	2368 ± 524
Protein (g)	88 ± 5	84 ± 5	163 ± 82	104 ± 29
Total Fat (g)	81 ± 5	78 ± 5	85 ± 10	100 ± 25
Carbohydrate (g)	221 ± 11	226 ± 12	270 ± 23	268 ± 46
Sugars, total (g)	77 ± 6	83 ± 6	97 ± 11	90 ± 18
Fiber, total dietary (g)	19 ± 1	20 ± 2	29 ± 4	25 ± 4
Added sugar (g)	42 ± 4	46 ± 8	50 ± 8	38 ± 12
Total Fruits (cup)	1.0 ± 0.1	1.0 ± 0.1	1.5 ± 0.3	1.5 ± 0.3
Total vegetable (cup)	1.9 ± 0.2	1.7 ± 0.2	1.7 ± 0.2	2.4 ± 0.4

* No significant changes in dietary pattern during the study periods

Table S4 Method validation for linearity, limit of detection (LOD) and limit of quantification (LOQ), precision (within-run and between-run), recovery and matrix effect using standards dissolved in starting mobile phase (SMF)[1] and in biological samples (plasma and urine).

Standards	Calibration range (nmol/L)	LOD (nmol/L)			LOQ (nmol/L)			Within-run precision (RSD%)[2]	Between-run precision (RSD%)[2]	Recovery (%)		Matrix effect (%)[3]	
		SMF	Plasma	Urine	SMF	Plasma	Urine			Plasma	Urine	Plasma	Urine
Cyanidin 3-O-glucoside	LOQ-140	0.1	0.1	0.1	0.3	0.6	0.6	0.3-7.8	1.3-5.1	74	76	-17	-31
Cyanidin 3-O-rutinoside	LOQ-430	0.1	0.1	0.1	0.2	0.2	0.2	0.1-1.1	2.2-4.3	76	57	-12	-32
Peonidin 3-O-glucoside	LOQ-69	0.03	0.03	0.1	0.1	0.3	0.3	0-2.8	0.9-1.9	71	44	-24	-41
Pelargonidin 3-O-glucoside	LOQ-74	0.1	0.1	0.1	0.1	0.3	0.3	2.2-3.9	3.3-4.1	77	62	-23	-39
Pelargonidin 3-O-rutinoside	LOQ-55	0.1	0.1	0.1	0.2	0.2	0.4	1.1-1.4	3.6-5.1	78	34	-17	-41
Urolithin A 3-O-glucuronide	LOQ-1267	2.5	2.5	2.5	9.9	9.9	9.9	1.7-1.8	0.3-3.1	106	84	-37	-28
Urolithin B 3-O-glucuronide	LOQ-660	0.7	1.3	1.3	2.6	2.6	2.6	0.5-2.5	2.7-3.4	83	61	-44	-58
Ellagic acid	LOQ-424	13.2	13.2	13.2	53	53	53	1.2-3.4	4.1-10.3	71	49	7	-36
Epicatechin	LOQ-44138	13.8	13.8	13.8	27.6	55.2	55.2	0.5-1.9	6.4-6.7	69	94	-22	-23
2-Hydroxybenzoic acid	LOQ-1855	3.6	7.2	3.6	7.2	14.5	7.2	0.7-1.6	3.1-4.4	71	99	-63	-96
3-Hydroxybenzoic acid	LOQ-7420	29.0	29.0	58.0	115.9	115.9	115.9	5.2-9.0	2.2-9.3	87	95	8	-56
4-Hydroxybenzoic Acid	LOQ-14841	7.2	29.0	58.0	29.0	58.0	115.9	0.6-6.2	7.5-7.9	124	95	7	-30
2,3-Dihydroxybenzoic acid	LOQ-3324	6.5	3.2	6.5	13.0	6.5	13.0	2.1-4.5	4.1-5.2	93	99	55	-16
2,5-Dihydroxybenzoic Acid	LOQ-3324	6.5	3.2	13.0	13.0	6.5	26.0	2.1-2.3	6.3-7.9	67	93	37	-66
3,4-Dihydroxybenzoic acid	LOQ-1662	6.5	6.5	13.0	13.0	13.0	26.0	2.6-3.4	0.7-5.8	95	97	9	-90
Vanillic acid	LOQ-195048	23.8	47.6	95.2	95.2	95.2	190.5	4.3-5.8	5.2-11.2	86	99	-8	-47
Syringic acid	LOQ-10343	40.4	40.4	161.6	161.6	80.8	323.2	2.1-2.9	7.7-12.7	80	104	-1	-75
4-Hydroxybenzaldehyde	LOQ-2098	2.0	4.1	8.2	4.1	8.2	16.4	2.4-3.0	2.6-5.3	95	91	11	1
3,4-Diydroxybenzaldehyde	LOQ-1855	0.9	0.9	7.2	1.8	1.8	14.5	3.0-8.8	5.5-10.0	53	97	36	-95
Phloroglucinaldehyde	LOQ-1662	1.6	1.6	1.6	3.2	3.2	3.2	1.9-24.3	0.2-10.8	69	99	-6	-32
Vanillin	LOQ-1684	6.6	6.6	13.2	13.2	13.2	26.3	1.8-7.3	3.6-12.6	90	102	-22	-63
p-Coumaric acid	LOQ-1561	1.5	3.0	3.0	6.1	6.1	6.1	2.1-3.5	3.6-8.5	88	101	-46	-63

m-Coumaric acid	LOQ-1561	12.2	12.2	12.2	24.4	24.4	24.4	2.3-6.7	1.7-5.8	92	101	-13	-47
o-Coumaric acid	LOQ-1561	3.0	3.0	3.0	6.1	6.1	6.1	0.2-0.4	0.8-2.3	89	97	-22	-65
Caffeic acid	LOQ-5689	22.2	22.2	22.2	44.4	44.4	44.4	0.7-2.0	0.7-7.6	88	102	-4	-31
Ferulic acid	LOQ-21113	5.2	5.2	5.2	10.3	10.3	10.3	0.8-17.6	7.0-11.7	82	95	-26	-20
Sinapic acid	LOQ-9143	8.9	8.9	8.9	35.7	35.7	35.7	2.0-2.3	1.3-2.1	55	93	5	-15
Hippuric acid	LOQ-91531	11.2	357.5	715.1	44.7	1430.2	2860.3	0.7-0.9	2.3-3.8	94	96	-34	-21
2-Methylhippuric Acid	LOQ-5306	41.5	41.5	82.9	82.9	82.9	165.8	1.1-4.0	3.1-4.2	100	88	-29	-5
4-Methylhippuric Acid	LOQ-5306	20.7	20.7	20.7	41.5	41.5	41.5	0.9-4.4	0.8-7.8	86	99	-47	-56
2-phenylacetic acid	LOQ-30118	941.2	941.2	941.2	1882.4	1882.4	1882.4	1.3-18.5	5.9-20.6	91	93	-21	-44
2-Hydroxyphenylacetic acid	LOQ-3368	6.6	13.2	13.2	26.3	26.3	26.3	1.2-1.6	3.9-4.3	78	94	19	-45
3-Hydroxyphenylacetic acid	LOQ-26947	52.6	52.6	105.3	210.5	210.5	210.5	1.4-3.0	2.4-6.1	108	98	-26	-52
4-Hydroxyphenylacetic acid	LOQ-107789	842.1	1684.2	3368.4	1684.2	3368.4	6736.8	1.2-6.3	8.8-9.0	83	99	-18	-77
4-methoxyphenylacetic acid	LOQ-3084	12.0	24.1	24.1	24.1	48.2	48.2	2.0-2.1	3.5-5.1	104	98	-15	-35
3, 4-dihydroxyphenylacetic acid	LOQ-48762	23.8	23.8	95.2	47.6	47.6	190.5	5.1-5.8	1.3-3.9	81	101	7	-86
Homovanillic acid	LOQ-180044	703.3	703.3	1406.6	1406.6	1406.6	2813.2	3.0-3.7	2.2-6.6	212	106	-3	-40
3-(3-Hydroxyphenyl)propionic acid	LOQ-3084	12.0	48.2	48.2	48.2	96.4	96.4	2.2-4.1	3.7-6.9	98	97	-12	-32
3-(4-Hydroxyphenyl)propionic acid	LOQ-98699	1542.2	6168.7	6168.7	6168.7	12337.4	12337.4	8.5-37.4	25.7-45.1	387	64	3	-81
Hydrocaffeic acid	LOQ-22505	87.9	175.8	175.8	175.8	351.6	351.6	1.3-1.6	3.9-5.3	66	96	-16	-78
Hydroferulic acid	LOQ-167184	20.4	40.8	81.6	81.6	81.6	163.3	4.8-7.9	11.3-33.2	81	101	-2	-34

[1]Starting mobile phase (SMF), water containing 1% formic acid and 5% acetonitrile

[2]Range of relative standard deviations (RSD %) calculated with the standards in SMF and in the different matrices.

[3]Matrix effect % = (slope calibration curve in matrix - slope calibration curve in SMF)/slope calibration curve in SMF × 100.
